# From Plantation to Cup: Changes in Bioactive Compounds during Coffee Processing

**DOI:** 10.3390/foods10112827

**Published:** 2021-11-17

**Authors:** Februadi Bastian, Olly Sanny Hutabarat, Andi Dirpan, Firzan Nainu, Harapan Harapan, Talha Bin Emran, Jesus Simal-Gandara

**Affiliations:** 1 Department of Agricultural Technology, Hasanuddin University, Makassar 90245, Indonesia; olly_hutabarat@unhas.ac.id (O.S.H.); dirpan@unhas.ac.id (A.D.); 2 Department of Pharmacy, Faculty of Pharmacy, Hasanuddin University, Makassar 90245, Indonesia; firzannainu@unhas.ac.id; 3 Medical Research Unit, School of Medicine, Universitas Syiah Kuala, Banda Aceh 23111, Indonesia; harapan@unsyiah.ac.id; 4 Department of Pharmacy, BGC Trust University Bangladesh, Chittagong 4381, Bangladesh; talhabmb@bgctub.ac.bd; 5 Nutrition and Bromatology Group, Department of Analytical Chemistry and Food Science, Faculty of Science, Universidade de Vigo, E32004 Ourense, Spain

**Keywords:** coffee, preharvest coffee, postharvest coffee, bioactive changes

## Abstract

Coffee is consumed not just for its flavor, but also for its health advantages. The quality of coffee beverages is affected by a number of elements and a series of processes, including: the environment, cultivation, post-harvest, fermentation, storage, roasting, and brewing to produce a cup of coffee. The chemical components of coffee beans alter throughout this procedure. The purpose of this article is to present information about changes in chemical components and bioactive compounds in coffee during preharvest and postharvest. The selection of the appropriate cherry maturity level is the first step in the coffee manufacturing process. The coffee cherry has specific flavor-precursor components and other chemical components that become raw materials in the fermentation process. During the fermentation process, there are not many changes in the phenolic or other bioactive components of coffee. Metabolites fermented by microbes diffuse into the seeds, which improves their quality. A germination process occurs during wet processing, which increases the quantity of amino acids, while the dry process induces an increase in non-protein amino acid γ-aminobutyric acid (GABA). In the roasting process, there is a change in the aroma precursors from the phenolic compounds, especially chlorogenic acid, amino acids, and sugars found in coffee beans, to produce a distinctive coffee taste.

## 1. Introduction

Coffee is one of the most important crops and is therefore of enormous economic relevance. The Genus Coffee belongs to the *Rubiaceae* family. While there are around 103 species globally, the two main coffee tree species cultivated on a worldwide scale are *Coffea arabica* (arabica) and *Coffea canephora* (robusta), representing 70% and 30% of the coffee market, respectively [1,2]. Coffee goes through a lengthy procedure before becoming a cup of coffee. Starting with the harvest, and progressing through fermenting, drying, and roasting, the coffee is transformed into a cup of coffee after brewing [3,4,5]. During this process, several physical and chemical changes occur. Coffee quality begins with the selection of uniformly matured coffee beans [5]. Differences in the level of maturity of coffee beans naturally can be found on the same tree. Therefore, when picking, only ripe fruit is picked for better coffee quality. Unripe coffee processing adds to a high number of faulty beans, resulting in a substantial volume of low-quality coffee [6,7,8]. Before picking, the coffee fruit should have adequate time to develop. This stage is the beginning of the formation of several components that play an essential role in the quality of coffee drinks [9].

During postharvest, several metabolic activities occur, depending on the type of processing carried out. The chemical composition of coffee beans can change due to physical, biochemical, and physiological changes during the postharvest and drying process. Any changes in these compounds result in varying precursors and affect the coffee beverage’s ultimate quality after roasting [6].

There are various categories of chemical compounds in coffee fruit that play a role in the production of taste and health benefits. Nitrogenous components (alkaloids, trigonelline, proteins, and free amino acids), carbohydrates, lipids, chlorogenic acids, organic acids, and volatile chemicals are among these groupings of compounds [9,10]. Some of these components are precursors to the flavor development of volatile and semi-volatile compounds in coffee during roasting. The group comprises acids, alcohols, aldehydes, esters, furans, indoles, ketones, phenolic compounds, pyrazines, pyridines, pyrroles, and thiols produced from around 900 volatile chemicals, which provide coffee with its flavor and aroma [4,11].

It is now well known that coffee is consumed not only for its distinctive flavor but also for its stimulating effect and its health benefits. Coffee’s health advantages are related to its high antioxidant activity, which is strongly affected by the total phenolic content (TPC) in coffee beans [4]. Although extensive reviews have been carried out on chemical constituent shifts during coffee processing, alteration of the bioactive compounds from plantation to cup is still not elucidated so far. This review presents some studies regarding the changes in bioactive compounds in coffee during preharvest and postharvest.

## 2. Parts and Chemical Compounds of Coffee Cherry Bean

An important factor in the relevance of caffeine for human health, the quality of coffee beverages is determined by changing the biochemical compounds during processing, and the development of coffee trees from seed, germination to harvest is one of the essential keys to coffee quality [12,13]. In addition, coffee trees grow well depending on cultivation areas, the influence of genotype, conservation, and other factors [14]. Furthermore, the climatic factor is known to be directly related to a volatile organic compound in green Arabica coffee beans and coffee beverage quality [15,16].

**Figure 1:**
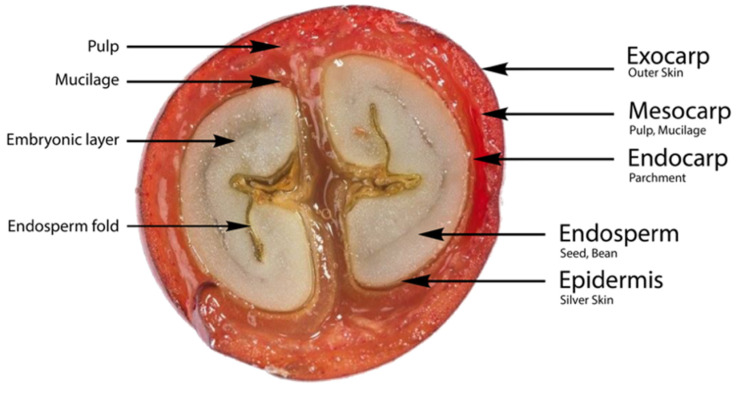
The anatomy of the cherry coffee bean.

An understanding of the alterations occurring in coffee fruit during development stages should start with the parts of coffee bean. Figure 1 below presents a basic illustration of coffee fruit tissue (Figure 1).

Coffee cherry fruit consists of an exocarp, mesocarp, endocarp, silver skin, and endosperm (bean). The outer skin, or exocarp, is also referred to as the peel, the outermost layer of the coffee fruit. It is formed by a single parenchyma cell, with the primary walls containing chloroplast, and can absorb water. The mesocarp, also known as the mucilage, is the flesh of the coffee fruit. In unripe coffee fruit, this tissue is stiff. The next part of the coffee fruit is the innermost layer of the pericarp. The endocarp is the hull that covers the coffee bean. It comprises three to seven layers of sclerenchyma cells, which act as primary assistance cells. The seed skin is the spermoderm, generally referred to as the silver skin, and the endosperm (seed) surrounds the embryo and supplies starch and may also contain proteins and oils [9,17].

Coffee plantations can be productive for up to 30 years. First flowering and fruit production occur roughly three years after germination, and fruit grows significantly 0–2 months after flowering. Development of perisperm occurs 2.5–3 months after flower, whereas endosperm continues to grow gradually for around five months after blooming [17].

The exocarp is part of the pericarp, which is active tissue in immature green coffee berries. During pericarp (exocarp, mesocarp, and endocarp) maturation, the chlorophyll, carotenoids, and anthocyanin compounds gradually decrease [9,17] Cherries may produce one-third of coffee trees’ daily respiration, and contribute around 12% of their total carbon requirements. This contribution is needed at the bean filling stage, as the beans are composed of carbohydrates. Determination of solid soluble content in various ripening stages showed the highest Brix degree value occurring in overripe fruits. The increase showed in the days after flowering [18].

The mesocarp is around 0.5–2 mm in thickness, and is divided into external and internal parts. The mesocarp is formed from compact parenchyma cells and composed of sugars, sucrose, and water. During the immature stage, cell walls are thicker than in the mature stages. The changes in thickness are due to the pectin modifications. The endocarp, called the parchment layer, has a hard and lignified tissue of around 0.15 mm. The endocarp protects the coffee bean from digesting enzymes.

The coffee perisperm, which has also been in the past referred to as the “spermoderm”, develops from the nucellus of the ovule soon after fertilization, zero to three months after flowering, cell-division occurring in the early stages. The perisperm emerges as a thinner pellicle at the mature stage, an approximately 0.7 mm silver skin formed by sclerenchyma cells arranged longitudinally. The thin pellicle is observed as a result of dehydration due to absorbed perisperm cells [9,17]. Due to the sporophytic origin of the perisperm, the activity of enzymes and gene expression in this tissue demonstrate that it contributes to the physical characterics of coffee beans, particularly when it is separated from the endosperm [19,20].

### 2.1. Maturity of Coffee Bean

A high-quality coffee beverage is affected by bean maturity. The accumulation of certain chemical compounds in mature coffee beans results in a flavorful beverage, and this objective plays an essential role in coffee processing [21,22]. Chlorophyll, also known as tetrapyrroles, makes plants green, allowing them to absorb blue and red light strongly. Chlorophyll a and chlorophyll b are the major types of chlorophylls found in plants [23]. The exocarps persevere as green tissue before maturation and gradually develop toward yellow and then red in the final stages of development [9,17]. The alteration of green to yellow is affected by carotenoids. Carotenoids play the predominant role in yellow pigmentation. However, in some instances, flavonoids are responsible for the color. More frequently, yellow flavonoids co-occur with carotenoids. The transformation of the coffee cherry from a yellow to a red color is due to the accumulation of high anthocyanin amount. The term anthocyanin was coined for the most important flavonoid pigment, which was first detected in the purple pigment in viola flowers. In some cultivars, the exocarp remains yellow. However, the shift in color is the most important factor, since fruit maturation is the reference point [23].

Yukiko Koshiro et al. [24] reported that of the five different stages of the coffee bean, in young fruit, fructose, glucose, and quinic acid were detected at high concentrations. In the later stages of development, sucrose was found to be significantly high. Sucrose in the ripened pericarp and seeds of cultivars of C. arabica was found to be higher than in *C. canephora*. However, much higher sucrose levels were reported in the seeds of cultivars of *C. arabica* than in those of *C. canephora*, whereas fructose and glucose accumulated in pericarps of all coffee samples and the seeds of *C. canephora*. During the development and ripening of fruits, alterations in malic acid, citric acid, oxalic acid, and quinic acid occurred. These are found in different concentrations in seeds and pericarps. Citric acid was at its highest amount in seeds, whereas malic acid was detected highest in pericarps. The color change of the pericarp comprises the last step in fruit development. Before this step, the first stage consists of tissue changes, after which a liquid state replaces the waxy state due to the accumulation of high levels of protein, sucrose, and polysaccharides [9,25].

Climate variations at different stages of maturation influence coffee quality. Many studies have already investigated the impact of environment on genotype bean transcriptome of the different ripening stages of the bean: immature (green), intermediate (yellow), and mature (red). The results showed that the yellow stage had a more significant transcript numbers [26]. Green coffee bean quality is affected by climatic factors, as shown in a study on whether high altitude growing contributed to volatile compounds in coffee crops compared to growing in lowland regions. Sixteen samples from highland and lowland locations were tested using GC–MS to analyze the group of volatile compounds. Samples from highland sites showed high concentrations of ethanal and acetone compounds. Meanwhile, samples from lowland sites showed high concentrations of alcohol, aldehyde, hydrocarbon, and ketone compounds. Furthermore, butane-1,3 diol, and butane-2,3 diol were detected in the lowland region, and were found to lead to off-flavors [16].

### 2.2. Bioactive Compound in Coffee Cherry Bean

The most abundant chemical in coffee is caffeine, which produces the strong aroma that makes coffee one of the world’s popular beverages. The fruit of the coffee tree is known as a cherry, and the beans that develop inside the cherry are used as the basic element for producing roasted, soluble coffee powders and coffee liquor. Caffeine and chlorogenic acid are detected at higher levels in robusta than in arabica coffee. Both caffeine and chlorogenic acid are responsible for bitterness [12,13,27].

Polyphenols chlorogenic acid and caffeoylquinic acid were detected in large amounts in green coffee beans. Tannins are detected in green coffee beans in the skin, while anthocyanins are found in the red fruit. An important characteristic of anthocyanins is that they undergo pH-dependent changes in color intensity and hue, or even loss of color. This is because an anthocyanin molecule undergoes complex rearrangements in aqueous solutions, existing in different-colored or colorless forms depending on the pH of its environment [23].

Caffeoylquinic acids, feruloylquinic acids, and dicaffeoylquinic acids are the main groups of chlorogenic acid reported in the coffee bean. Chlorogenic acid is the main component of phenolic green coffee beans, formed by one molecule of quinic acid and one to three molecules of trans-hydroxycinnamic acid [28]. Chlorogenic acids are distributed in large plant kingdoms, but green coffee beans contain the most abundant levels. Lower levels of compounds of chlorogenic acid have also been observed in species from Africa compared to other regions. Small amounts of compounds of chlorogenic acid were identified as di-feruloyl quinic acids, dimethoxycinamoylquinic acids, caffeoyl-dimethoxycinamoylquinic acids, and feruloyl-dimethoxycinamoylquinic acids in robusta. Furthermore, in coffee pulp, chlorogenic acid–protein complexes were found in immature coffee beans. Using chromatography, nine isomers of chlorogenic acid were identified in abundant levels in green coffee beans [27,29].

Chlorogenic acid is observed in higher amounts in immature coffee beans than in ripened beans, which present with total chlorogenic acid levels 6.5-fold lower than immature beans. Hydrolysis of dicaffeoylqunic acid into monoester causes an increasing ratio of caffeoylquinic acid/dicaffeoylquinic acid during the maturation stage until the fruit reaches ripeness. However, before ripening, caffeoylquinic acid contents start to drop, probably due to oxidation. The same results were confirmed a previous study [28] that found that feruloylquinic acid and dicaffeoylquinic acid levels decreased significantly at the last step of maturation. The larger amount of chlorogenic acid content in immature beans than in mature fruit is related to the high polyphenol oxidase and peroxidase levels in immature stages compared to the mature stage. According to these results, ripe seeds have lower sensitivity to oxidases than unripe seeds, due to decreased levels of both polyphenol oxidase, an enzyme that plays an essential role in response to pest attack, and peroxidase, which is an important enzyme in scavenging reaction oxygen [27,30].

PPO is found in most plants. Most PPOs are believed to be localized in the chloroplast. Chloroplast transit peptides and thylakoid transfer domains can be removed by proteolytic processing. They are closely related to tyrosinase and located in the chloroplasts. The PPO gene is encoded in the nucleus and translated into the cytoplasm. The pro-PPO formed is then transported to the chloroplast. PPO is generally bound to the thylakoid membrane [31]. The conversion of phenolic substrate to o-quinones occurs in two reactions, namely, hydroxylation and oxidation [27,30]. Hydroxylation is the first step in oxidation by polyphenol oxidases (PPO), which results in highly reactive quinoid structures that can interfere with cell-invading pathogens [32]. The hydroxylation pattern influences physiological properties, such as light absorption and antioxidative activity, which are the basis of the beneficial health effects of flavonoids [33].

Chlorogenic acids are abundant in immature coffee cherries [28] due to PPO. PPO activity in the coffee plant is considered intrinsic to the quality of the coffee beverage. The activity of PPO in leaves and in the development of the endosperm was determined [34]. Polyphenol oxidation, particularly chlorogenic acids, leads to the forming of quinones, which can affect the taste and nutritional qualities of the coffee beverage. PPOs are also involved in plant disease-resistance. Quinones may also react with amino acids and protein, which indicates that PPO activity is closely related to the quality of the coffee beverage [35]. This was also confirmed by Chen et al. [36] that the enzyme-catalyzed browning reaction involves oxidation of the phenolic compound into quinones mediated by the enzyme PPO, followed by the transformation of the quinones to form the dark pigment. It leads to deterioration of flavor, color, and nutritional quality, occurring throughout the process, from harvest handling to the marketing of the produce.

Membrane damage could inhibit the increase in quinones from the oxidation of chlorogenic acids, thereby contributing to a quality cup [37]. The PPO activity in leaves and the development of endosperm showed high enzymatic activities in the studies reviewed. PPO activity was detected at higher levels in young leaves than mature leaves, whereas activity in endosperm development was lower approximately 100 days after flowering, then significantly increased around 170 days after flowering and gradually decreased in mature beans. PPO activities followed the synthesis of chlorogenic acids in both leaves and endosperm [34]. PPO is sensitive to temperature. The enzymatic reaction rate increases with increasing temperature to a maximum level, and then decreases with further temperature increase due to enzyme denaturation [38,39].

Besides chlorogenic acid, caffeic acid is also oxidized by PPO. It was detected that caffeic acid leads to the formation of caffeoquinone, which reacts with glycine methyl ester to produce a green color. The reaction of the quinones with free amino acids, peptides, or proteins results in a blue pigment that becomes a green pigment in the presence of excess caffeoquinone [35,36,37,38,39]. The color product can impact the taste of the coffee.

While it is important to understand the roles of bioactive compounds during fruit development, the coffee bean transcriptome explains the accumulation of the major bean components through ripening [26]. The composition of the maturing coffee bean determines the processing performance and ultimate quality of the coffee produced from the bean. Analysis of the differences in gene expression during bean maturation may explain the basis of genetic and environmental variation in coffee quality. The transcriptome of the coffee bean was analyzed at three stages of development, namely, immature (green), intermediate (yellow), and mature (red) [26].

Besides chlorogenic acid and caffeine, tryptophan is one of the major bioactive compounds in coffee. Caffeine (1,3,7-trimethylxanthine), a purine alkaloid, is a popular compound in coffee beverages and is distributed in a few plant products [40]. Caffeine was found in many parts of the coffee plant, particularly in leaves and cotyledons, in different concentrations. It was approximately less than 1%–2% of dry weight. However, in roots and older brown parts and shoots, no caffeine was observed. Extensively investigated, the caffeine content of distinct varieties of coffee was also determined, and the result showed that the highest amount of caffeine was recorded in *Coffea canephora* (robusta). Caffeine synthesis occurs in leaves, perisperm, and pericarp during fruit development. The biosynthesis of caffeine is composed mainly of three methylations and one nucleosidase reaction [41]. A similar finding by Perrois et al. [42] confirmed that the gene-expression differential regulation of caffeine metabolism in coffee arabica and robusta showed higher caffeine content in robusta than in arabica. Compared to the various parts of coffee, caffeine accumulation is higher in the leaves than in the coffee bean during the maturation period. The differences in concentration in these two species indicate different genes encoding N-methyltransferase [42]. In leaves, the biosynthesis of caffeine takes place during leaf emergence, and in this stage, the caffeine compound was detected at its most abundant levels in young leaves, whereas in the pericarp and perisperm it was found in lower amounts. However, the concentrations of methionine in the pericarp were found to be twenty-five-fold higher than in the perisperm. During fruit development, the higher caffeine content reached the seeds between eight and twelve weeks after flowering, and decreased gradually after sixteen weeks. In this stage, the caffeine content detected was constant in the pericarp, showing the cessation of caffeine biosynthesis due to its actively proceeding to the bean [9].

Comai et al. [43] reported that tryptophan is a minor amino acid in the human diet. However, green coffee provides the most abundant free amino acids. Therefore, green coffee is highly recommended as a free and protein-bound tryptophan [44]. Tryptophan has various biological effects on the body, including altering mood, cognition, sleep, aggressiveness, vomiting, and other physiological processes. [45]. Tryptophan and 5-hydroxytryptophan (5-HTP) are present in green and roasted coffee [44]. Decreasing the temperature during the roasting process may form serotonin [46]. Mood, the immune system, cardiovascular disease, and other conditions could be affected by drinking coffee due to the tryptophan content. In addition, coffee extracts and some of their compounds suppress the breakdown of tryptophan and neopterin production and are anti-inflammatory and immunosuppressive. It was confirmed in vivo that consuming coffee increases the availability of neurotransmitter 5-hydroxytryptamine (serotonin), which can improve mood and quality of life [46].

The highest oxygen radical absorbance capacity (ORAC) values indicated that coffee extracts efficiently scavenge radicals, therefore suppressing tryptophan breakdown in mitogen-stimulated peripheral blood mononuclear cells [46]. Total tryptophan and 5-hydroxytryptophan (5-HTP) on green beans of arabica and robusta were determined. The results showed that the protein tryptophan was found in larger amounts in robusta than arabica. However, free tryptophan levels were detected at much higher levels in arabica than in robusta. Roasting may reduce the protein tryptophan. The Brazilian medium-roasted coffee beverage consists of 1.4–2.5 mg tryptophan/50 mL cup [47].

Tryptophan plays an essential role during the growth and development of leaves, fruits, and seeds and is involved in protein biosynthesis. One study found that large amount of tryptophan in immature beans could be converted into auxin [22]. Nine different varieties of green Coffea arabica beans in different stages of development were analyzed using LC-MS. The results showed a strong relationship between maturity and metabolome. According to the partial least square results, tryptophan was detected at much higher levels in immature beans than in the semi-mature, mature, and overripe stages, and this contributed to the regression model. These findings confirm that tryptophan is a specific marker of immaturity of coffee. Tryptophan is used as a marker of immaturity, but also in practical cases, the amount of tryptophan should be assessed to ascertain the quality of green arabica coffee bean [22]. The results suggest that traders could use tryptophan as a standard for certified trading of green arabica beans. Furthermore, the results showed no close relation between tryptophan metabolism and the development of coffee beans.

### 2.3. Lipids, Proteins, and Carbohydrates in Cherry Coffee Bean

Lipid formation at the green cherry stage was found to be extremely high expression compared to the yellow stage. In addition, alpha-galactosidase was detected at levels ten-fold lower in the yellow stage than in the green stage. Furthermore, proteins reached higher levels in the green stage, but significantly decreased at the yellow and red stages. Lipids are stored in triacylglycerol and linoleic acid (fatty acid desaturation), while proteins are identified as globulins [26].

Coffee beans are composed of cell wall polysaccharides, lipids, proteins, sucrose, and chlorogenic acids. In order to support the growth of embryos and reproduction, some nutrients are needed. Nutrients are formed with complex pathways in beans [25]. The more comprehensive range of green coffee beans correlated with the storage phase, when most components are formed. One of the compounds stored is galactomannan, which can react with proteins through Millard reactions. These reactions contribute to coffee aroma, and influence the viscosity of the coffee beverage. Galactomannan is produced not only during the processing of the coffee beverage, but also in the plant itself through osmotic stress, when microbial attacks are affected by the high viscosity and low solubility of galactomannan [25]. Globulins, such as glutelin and patatin, are the most major storage proteins in coffee. Approximately one-third of the total proteins are formed late in bean development. However, triacylglycerol and linoleic acid gradually decrease through ripening. The end of bean maturity is denoted by more cell-wall degradation and higher numbers of transcript expressions at the yellow stage, and lower numbers of transcript expressions and less cell-wall degradation in the red stage. These phenomena indicate bean maturity and minor changes in the coffee when the pericarp changes from yellow to red [26].

## 3. Harvesting of Coffee Beans

The quality of coffee beverages is affected by the length of fruit maturation. Climatic conditions, such as air temperature, evapotranspiration, rainy season or dry season affect the timing of the harvest. Some cultivars of coffee from different regions in Brazil were evaluated regarding the correlation between length of maturation and chemical compounds. In cooler regions, ripening is generally later and will produce flavorful beans due to the longer cycle involved in the formation of the bioactive compounds. However, tryptophan and chlorogenic acid do not form properly in a short cycle, and these compounds give a bitter taste to the beverage. Fully mature fruit and knowing the time of harvesting is pivotal, resulting in better characteristics for the coffee beverage [48].

The different levels of amino acids in coffee are affected by several factors: species, variety, degree of ripening, post-harvest conditions, and analysis method. Coffee grown in a high air temperature is analyzed earlier, around two to three months, while coffee grown in lower air temperature is analyzed later. Another factor that causes coffee maturation is the availability of water, therefore in high air temperatures, evapotranspiration coffee matures faster than in low air temperatures [49].

The changes in the color of cherries are an indicator of the level of maturation. Green cherries are recognized as unripe, whereas black cherries are riper than grayish-red or yellow fruit. Unripe cherries are firmer than ripe cherries. Therefore, ripe fruit is susceptible to mechanical damage. Chemical contents change at the last stage of ripening, for instance, chlorophyll degradation, synthesis of carotenoid, and the development of anthocyanins during the transformation from yellow to red. Volatile compounds, esters, aldehydes, ketones, and alcohols increase, and astringency decreases at the end of maturation. These compounds contribute to the aroma of mature fruits [48].

In order to obtain a high-quality coffee beverage, green, red, partly ripe, and black cherries are not recommended for mixing; black berries, in particular, produce a woody flavor. The ripe—red or yellow—fruit are harvested first, while the green fruits should be picked at the end of the harvest. Harvesting cherries should be conducted as quickly as possible to avoid the negative effects of loss of freshness [17]. High-quality coffee is obtained from fresh, ripe cherries, which are used as raw materials from different picking methods combined with various processing techniques [17]. Wet or semi-dry coffee-processing methods are used, depending on the ripeness of the cherries harvested. However, collecting all fresh ripe cherries can damage the tree. In order to sustain full cherry harvesting, selective hand-picking is recommended.

Selective harvesting and stripping are the most manual harvesting techniques. Selective harvesting is usually performed by pickers by holding their baskets at their waists. A high ratio of ripe to unripe fruit and long harvesting time should be avoided. Plastic sheets, canvas, or cloth spread under the coffee tree are usually used for the milking or stripping harvesting method, where all ripe fruit are collected. Major damage can occur if collected coffee fruit lies too long in dry weather during harvesting. There are two alternative technologies for mechanical harvesting. First, self-propelled machines usually apply to coffee plantations with middle slopes and wide spacing between the rows of trees. The machines are equipped with two shaking heads, a fish plate, and a pneumatic separator. The second mechanical method is a tractor-driven machine that has one single shaking head. The tractor-driven machines can be used on steeper slopes and are closer than self-propelled machines [17].

A hand-held picking machine is composed of two sets of rods. Two sets of rods transmit vibrations to the branches and cause ripe cherries to fall. A hand-held pneumatic harvester has two to six hand-held harvesting tools connected to the compressor and driven by a tractor. A hand-held harvester can be used in all conditions [17]. Compared to other methods, manual picking can be time-consuming and expensive in places where the required workforce is not readily available [50].

## 4. Mode of Processing Affects Coffee Quality

After harvesting, ripe coffee cherries go through many processes to become green coffee beans. Coffee cherries must be processed as quickly as possible to avoid spoilage or unwanted mold growth [51]. In general, three types of coffee processing procedures are used to obtain green coffee beans, i.e., wet, dry, and semi-dry methods. These processing methods aim to remove silver skin, parchment, mucilage, pulp, and skin to produce the green beans.

### 4.1. Dry Process/Natural Process/Sun-Dried

The dry process is the easiest technique for producing green coffee beans, but high-quality coffee is difficult to obtain. This method is applied by 80% of Yemeni farmers and 60% of farmers in Brazil and Ethiopia, and it mostly uses robusta coffee [52]. This approach produces a coffee with a fruity and cherry-like flavor.

The coffee cherries are instantly set to dry with sunshine in dry processing. Depending on the weather, with a cherry water-content of approximately 65%–70% [53,54], the drying process might take up to 2–4 weeks. Coffee beans are dried until reaching a water-content level of approximately 15%. When using a huller machine, it is difficult to hull the coffee beans if the moisture level is still high. However, if the moisture level is too low, it will fracture the coffee beans. After being separated, the coffee beans are dried again until they have a moisture content of 12% [55,56].

During the dry process, the metabolism of the coffee beans continues. In contrast to the wet method, in which the beans undergo a germination process, in the dry method, germination is inhibited [57]. In addition to germination, coffee cherries undergo other metabolic processes during postharvest processing. Table 1 shows the accumulation of γ-aminobutyric acid (GABA) in the dry process, whereas only small amounts are accumulated in the wet process [58,59].

**Figure 2:**
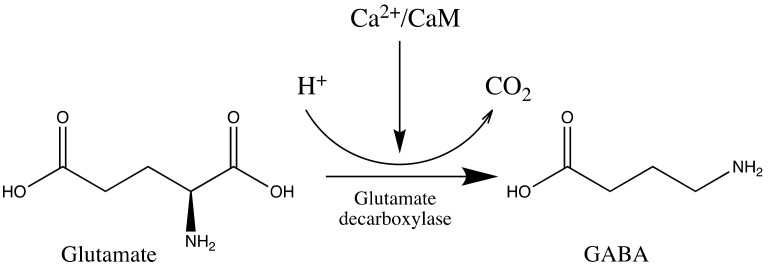
γ-aminobutyric acid (GABA) is produced by glutamate decarboxylate of glutamate.

In plants, GABA is one of the stress metabolites, and its accumulation is an indication that drought stress has occurred during the drying process. GABA is synthesized in plants by the pyridoxal 50-phosphate-dependent glutamate decarboxylase through the α-decarboxylation process of glutamic acid, in which either calcium/calmodulin or H^+^ quickly activates this enzyme (Figure 2) [60]. In recent decades, GABA has attracted much interest because of its numerous physiological effects on microorganisms, animals, and plants. In animals, GABA has a role in neurotransmission, neurodegenerative diseases, sleep and insomnia, and has antidiabetic, hypotensive, antidepression and anti-anxiety properties, among many other health advantages [60,61,62]. In addition to GABA accumulation, the presence of dehydrins, proteins that play a role in drought tolerance until metabolism stops due to low water content, is also an indication of drought stress [63,64].

The water content of the coffee beans decreases dramatically throughout the drying process. This condition occurs during both the dry and wet processes. However, drought stress only occurs in the dry process because it takes longer to dry the cherries. Meanwhile, the time necessary for the wet process is merely 2–4 days. Drought stress starts at 45% moisture, and metabolism shuts down at 25% moisture. The time required to reduce the moisture content to 25% in the wet process is relatively quick, resulting in less GABA. While the drying period of the dry procedure is longer than the wet process, the drought stress is likewise longer, and more GABA is produced [59].

### 4.2. Wet Process/Washed/Fully-Washed

Wet processing involves the mechanical depulping of coffee cherries, which involves squeezing off the majority of the fruit flesh. However, significant amounts of fruit flesh remain attached to the parchment. During the depulping process, the unripe fruit is separated from the ripe fruit, but the presence of some unripe fruits is unavoidable [65]. Fermentation degrades these mucilaginous residues, and then the resultant parchment coffee is dried to produce the green coffee bean [56,66,67]. This method was originally widely used in arabica coffee, but currently, many are using robusta coffee. The wet process produces coffee with more flavor and pleasant acidity, but with less body than the dry method [54,55,68] It was formerly assumed that this was because wet-processed coffee used ripe fruit, whereas dry-processed coffee paid less regard to fruit ripeness consistency [54]. However, the results of Valio’s research [57] show that although coffee cherries may have the same maturity level, the characteristics of coffee produced by dry processing and wet processing remain different. This is related to the germination process, which occurs immediately after depulping during the wet process.

Coffee beans are different from the seeds of legumes and cereals, which do not undergo a period of dormancy. The moisture content of ripe coffee cherries is 65% [53]. The water content in coffee cherries might allow the germination process to begin, however, the germination process is inhibited as long as coffee beans are covered with pulp. This is caused by a germination-inhibitor compound, abscisic acid [57]. This germination-inhibition process also occurs in some tropical fruits such as cacao, inhibited by high osmotic potential [69], and in tomatoes and avocados, inhibited by germination inhibitors and phytohormones [70,71].

The beginning of the germination process in seeds containing high fat levels is characterized by the presence of isocitrate lysine (ICL). These enzymes are germination-specific enzymes that are responsible for glyoxylate-cycle metabolism. ICL has a role in converting fatty acids into carbohydrates that occur at the transition stage from late embryogenesis to germination [64]. Research conducted by Selmar et al. [72] showed that significant ICL expression was detected in wet-processed seeds on the first day of processing. When the coffee fermentation phase was finished on the second day, the highest expression was visible and decreased during the drying process. In contrast, the maximum expression level was observed in dry-processed coffee seeds approximately one week after processing began [72,73]. The germination process causes the coffee beans to continue their metabolic process and produce amino acids that become flavor precursors [57]. Furthermore, the higher content of several free amino acids (glutamate, aspartate, and alanine) in wet processing is related to the protein breakdown that creates substrates for the germination process [58,74]. The difference in the number of free amino acids between wet and dry-processed coffee ranged from 3.3–20.9% [59].

### 4.3. Semi-Dry Processing/Honey-Coffee

Semi-dry processing combines the dry and wet process, in which the coffee fruits are mechanically pulped and proceed to dry without removal of the mucilage [75,76]. This method is known as the honey process, because the mucilage is dried along with the coffee beans and produces a honey-like or sugar-like aroma after the drying process. This method employs a depulper machine to remove the skin and pulp, leaving variable quantities of mucilage ranging from 20% to 80%. The mucilage composition consists of primary carbohydrates, i.e., polysaccharides (non-cellulosic and cellulose), pectin, and monosaccharides (glucose, mannose, xylose, arabinose, galactose, fructose, rhamnose, and uronic acid) which causes the smell of sugar in the semi-dry process coffee [77]. Chlorogenic acid is an antioxidant and an aroma precursor responsible for the bitter taste of coffee and is found in lower concentrations in the semi-dry process than in the dry process. In addition, the semi-dry method has less trigonelline than the wet and dry processes. However, the sucrose content is higher in the semi-dry process than in either the dry or wet processes. Therefore, the quality of semi-dry coffee may be described as being in between the quality of dry and wet procedures, and it is often used in espresso blends [78].

**Table 1:** Chemical content in various types of coffee processed in various countries.

Component	Coffee Sample	Processing Type	Value	References
Caffeine	Arabica, Brazil	Wet process	1.05–1.53%	[56]
	Arabica, Brazil	Wet process	1.32–1.42%	[79]
	Arabica, Thailand	Wet process	1.20–1.26%	[55]
	Robusta, Indonesia	Wet process	1.82%	[80]
	Arabica, Brazil	Dry process	1.24–1.35%	[79]
	Robusta, Indonesia	Dry process	1.81%	[80]
	Robusta, China	Dry process	1.88–2.61%	[81]
	Arabica, Brazil	Semi-dry process	1.12–1.54%	[56]
Trigonelline	Arabica, Brazil	Wet process	0.80–1.40%	[56]
	Arabica, Brazil	Wet process	1.01–1.18%	[79]
	Arabica, Brazil	Dry process	0.96–1.01%	[79]
	Robusta, China	Dry process	0.75–0.87%	[81]
	Arabica, Brazil	Semi-dry process	0.64–0.92	[56]
Chlorogenic acid	Arabica, Brazil	Wet process	6.08%	[56]
	Arabica, Brazil	Wet process	7.53–7.58%	[79]
	Robusta, Indonesia	Wet process	5.84%	[80]
	Arabica, Colombia	Wet process	4.89%	[82]
	Arabica, Brazil	Dry process	7.34–7.60%	[79]
	Robusta, Indonesia	Dry process	9.57%	[80]
	Arabica, Brazil	Semi-dry process	5.8%	[56]
	Arabica, Colombia	Semi-dry process	5.04%	[82]
Total Protein	Robusta, China	Dry process	15.3–16.4%	[81]
Phenolic compounds	Arabica, Brazil	Dry Process	6.9–7.6%	[83]
	Arabica, Brazil	Semi-dry process	7.67%	[84]
Free amino acid	Arabica, Brazil	Dry process	0.36%	[85]
	Arabica, Brazil	Wet Process	0.43%	[85]
	Arabica, Columbia	Dry process	0.27%	[85]
	Arabica, Columbia	Wet process	0.31%	[85]
	Arabica, Germany	Dry process	0.51%	[85]
	Arabica, Germany	Wet process	0.54%	[85]
	Arabica, Germany	Wet process	0.27–0.48%	[86]
	Robusta, Germany	Wet process	0.35–0.60%	[86]
GABA	Arabica, Brazil	Wet Process	93 nmol/seed	[58]
	Arabica, Tanzania	Wet Process	140 nmol/seed	[58]
	Arabica, Brazil	Dry Process	1009 nmol/seed	[58]
	Arabica, Tanzania	Dry Process	1860 nmol/seed	[58]
Carbohydrates				
Sucrose	Arabica, Brazil	Wet process	9%	[56]
	Arabica, Brazil	Wet process	±7.90%	[87]
	Arabica, Brazil	Wet process	5.89–7.31%	[79]
	Arabica, Kenya	Wet Process	9.31%	[87]
	Arabica, Costa Rica	Wet process	6%	[88]
	Arabica, Thailand	Wet process	4.43–4.85%	[55]
	Arabica, Brazil	Dry process	7.07%	[87]
	Arabica, Ethiopia	Dry process	8.26%	[87]
	Arabica, Brazil	Dry process	6.81–8.95%	[79]
	Robusta, Indonesia	Dry process	4.85%	[87]
	Robusta, Vietnam	Dry process	3.15%	[87]
	Robusta, Uganda	Dry process	4.56%	[87]
	Arabica, Brazil	Semi-dry process	±8.10%	[87]
	Arabica, Brazil	Semi-dry process	12.3%	[56]
Glucose	Arabica, Costa Rica	Wet process	0.02%	[88]
	Arabica, Brazil	Dry process	0.23%	[87]
	Arabica, Brazil	Wet process	±0.03%	[87]
	Arabica, Brazil	Semi-dry process	±0.11%	[87]
Fructose	Arabica, Costa Rica	Wet process	0.03%	[88]
	Arabica, Brazil	Dry process	±0.33%	[87]
	Arabica, Brazil	Wet process	±0.04%	[87]
	Arabica, Brazil	Semi-dry process	±0.19%	[87]
Total pectin	Arabica, Brazil	Dry process	899.09 mg/100 g	[83]
	Arabica, Brazil	Dry process	1191.81 mg/100 g	[84]
Lipid	Robusta, China	Dry process	8.60–12.03%	[81]

## 5. Microbiota Associated with Coffee Processing

### 5.1. Microbiota in Dry-Process

Naturally, microorganisms are present in all coffee processing, from harvesting until drying, and might impact the final beverage quality. Bacteria (gram-negative and positive), filamentous fungi, and yeast are abundant throughout the fermentation process (Table 2). Microbes that grow during the coffee fermentation process can come from the soil, water, air, handling at harvest, rainwater, fruit surfaces, agricultural equipment, and fermentation sites [75]. Although its chemical composition and aroma-forming precursors determine the characteristics of coffee, the microbiota also plays a role in sensory attributes. Fermented coffee has a high concentration of volatile compounds, such as alcohol, ketones, acids, esters, aldehydes, and other acceptable aroma compounds.

Meanwhile, unfermented coffee has lower volatile compounds and emits an unpleasant sulfur odor [93]. Microbes release metabolites throughout the fermentation process, which disperse into the beans. However, most studies (Table 3) focus on the function of microorganisms in mucilage degradation during processing [83,84,89,90,92]. Therefore, the microbial population is higher in the dry process than in the wet or semi-dry processes [83]. In addition, some research also investigated the role of microbes concerning the production of organic acids [92] and their ability to prevent the growth of ochratoxigenic fungi [94]. However, the actual role of each microbial group is unclear.

The populations of microorganisms are dependent on the processing method. In studies on the dry method, the population of bacteria is greater at the beginning, then yeast is at its highest at the 8th day, and the population of filamentous fungi is higher towards the final stages of the fermentation [83,89,96]. The significant number of bacteria at the first week of fermentation indicated the presented 70% water content and 0.85 a_w_ (activity water) of the coffee cherry, which continued to decrease until the end of fermentation [83]. *Bacillus* was the predominant species during dry processing [75,83,89]. These bacteria can be found in soil and secrete several enzymes, such as amylase, cellulase, and pectinase (polygalacturonase, pectic lyase, pectin methylesterase) that play a role during fermentation. In the first 12 h of fermentation, there is an increase in sugar [75]. This increase is due to the activity of microorganisms that convert pulp and mucilage into sugars. The good flavor of dry-processed coffee can be produced by the fermentation of lactic acid and acetic acid. Lactic acid production occurs under aerobic conditions, or due to ethanol oxidation by yeast or lactic acid bacteria. In the dry process, *Leuconostoc mesenteroides* bacteria are microorganisms that play a role in forming lactic acid [92]. During the fermentation process, there is a change in the composition of the pulp and mucilage caused by the endogenous metabolic activity of beans and the metabolic activity of bacteria. This change causes a decrease in pH from 6.5 to 5.5–5.8, and in water activity from 0.9 to 0.7–0.8, allowing yeast growth.

*Debaryomyces*, *Candida,* and *Pichia* were the predominant yeasts isolated from the dry process. These yeasts reached their maximum population on days 14–18 with a value of approximately 10^6^ cfu/g [83,104]. Species from the genus *Debaryomyces*, *Pichia*, and *Arxula* secreted pectinolytic enzymes that degraded pectin in pulp and mucilage.

### 5.2. Microbiota in Wet Process

During the wet process, the coffee cherries are pulped, fermented for 6–48 h, then washed to remove the remaining mucilage and dried. The wet process is mainly used for arabica coffee. Due to the shorter fermentation time, the number of microbes involved during the fermentation process is less than the dry process. In the wet process, water, time, and other conditions are more controlled to minimize the growth of unwanted microbes.

The isolation of bacteria in the wet process has been conducted since 1945. Several studies have succeeded in identifying groups of bacteria that have been isolated, such as *Lactobacillus*, *Streptococcus*, *Pseudomonas*, *Flavobacterium*, *Proteus*, *Paracolobactrum*, *Escherichia*, *Klebsiella*, *Weissela*, *Leuconostoc*, *Lactococcus*, *Acinetobacter*, *Enterobacter*, *Brevibacillus*, *Acetobacter*, *Asaia*, *Serratia*, and *Gluconobacter* [90,91,99,104,105]. Among these groups of bacteria, *Lactococcus*, *Leuconostoc*, *Acinetobacter*, and *Enterobacter* are predominant [90,91].

An increase in sucrose occurred at 8 h of fermentation, then decreased during fermentation, followed by a rise in organic acids after 24 h (malic acid, lysine, citric acid, arginine, isoleucine). Then fructose, glucose, gluconic acid, and leucine increased after 36 h of fermentation [90]. Sucrose levels are reduced due to the endogenous invertase enzyme’s activity, which causes the sugar to be broken down into fructose and glucose. Meanwhile, mannitol, lactic acid, and glycerol accumulation are produced by bacterial metabolism, especially lactic acid bacteria, *Leuconostoc mesenteroides*, through the fructose metabolic pathway. Several studies reported no effect on changes in plant-relative metabolite compounds in coffee, such as caffeine and trigonelline [52,85], but Zhang et al. [90] reported a decrease in caffeine and trigonelline at the end of the demucilage wet-process fermentation.

*Hanseniaspora*, *Candida*, and *Pichia* are the predominant species whose populations increase during the wet process. At the beginning of 24 h of fermentation, *Nectriaceae* was the predominant yeast. In addition, some species of *Penicillium*, *Neodevriesia*, *Aspergillus*, *Candida*, and *Cladosporium* are also only present at the beginning of fermentation [91]. In some fermentations, such as the fermentation of wine, cheese, bread, and cocoa, *Pichia kudriavzevii* is known to play a role in forming the aroma profile [92,99,105]. The role of yeast in degrading pectin in wet-process fermentation is similar to dry process fermentation. The isolated yeast had high pectinolytic enzyme activity to degrade pectin in pulp and mucilage. In addition, yeast also produces several flavor metabolites, such as aldehydes, some organic acids, ketones, esters, and alcohol [97].

### 5.3. Microbiota in Semi-Dry Process

The semi-dry process is a fermentation method between the dry process and the wet process. A depulper machine separates the ripe coffee fruit from the pulp, then the remaining coffee beans and mucilages are fermented and dried until moisture content decreases to 12%. Several studies have been conducted to identify the microbes present during the semi-dry fermentation process [84,92]. A previous study reported that *Escherichia coli*, *Lactobacillus Plantarum*, *Enterobacter agglomerans*, *Bacillus megaterium*, *Bacillus cereus*, and *Bacillus macerans* were the predominant bacteria during the semi-dry fermentation process [84]. *Bacillus* species were also identified as the higher populations during the semi-dry fermentation process by Velmourougane et al. [106]. *Pichia* species are the predominant yeast in the semi-dry process. It can be said that *Pichia* species are the predominant yeast in coffee fermentation because *Pichia* species were also the predominant yeast in dry and wet processes. Other yeast species present in the dry, wet, and semi-dry process include *Saccharomyces cerevisiae* and *Candida,* but in lower populations than *Pichia* [95,96]. In the semi-dry process, *Pichia anomala* is the only yeast species present from the point of harvest to the end of the fermentation. During the semi-dry process, other species yeasts were also discovered, including *Arxula*, *Candida*, *Saccharomyces*, *Debaryomyces*, *Mitchella*, *Trichosporon*, *Rhodotorula*, and *Torulaspora* [84,92].

## 6. Green Bean Storage

After processing, storage is a factor that influences beverage quality. Environmental factors, physical conditions, and time all have an impact on storage. According to Selmar et al. [74], green coffee beans should be stored without hulling at 22 °C and RH 63. Storage at temperatures above 40 °C causes cell damage due to leakage of intercellular materials in green coffee beans. Embryo seeds can continue to develop for up to 6 months; humidity (RH) conditions during storage must be addressed. After the death of the embryo, if seed moisture is above 20%, it will trigger the activity of enzymes in the seed, such as polyphenol oxidase (PPO) and lactase. These enzymes can oxidize phenolic compounds, such as chlorogenic acid, which is a flavor precursor during roasting. [74,78].

Green coffee beans can be stored for three years [78]. Generally, green coffee beans are stored using jute sacks because they are cheaper and can be reused. The use of a jute sack is recommended for storage of fewer than six months. Compared with storage using HDPA plastic, the total phenolic content and chlorogenic acid content in coffee stored using jute sacks decreased sharply at month ten, and there was a significant difference in storage for 11 months [74]. Enzymatic and non-enzymatic processes can cause a decrease in chlorogenic acid during storage [65]. Research conducted by Selmar et al. [74] recommends using HDPA plastic for coffee storage for more than six months, because of its ability to stabilize water content and color.

## 7. Roasting

**Figure 3:**
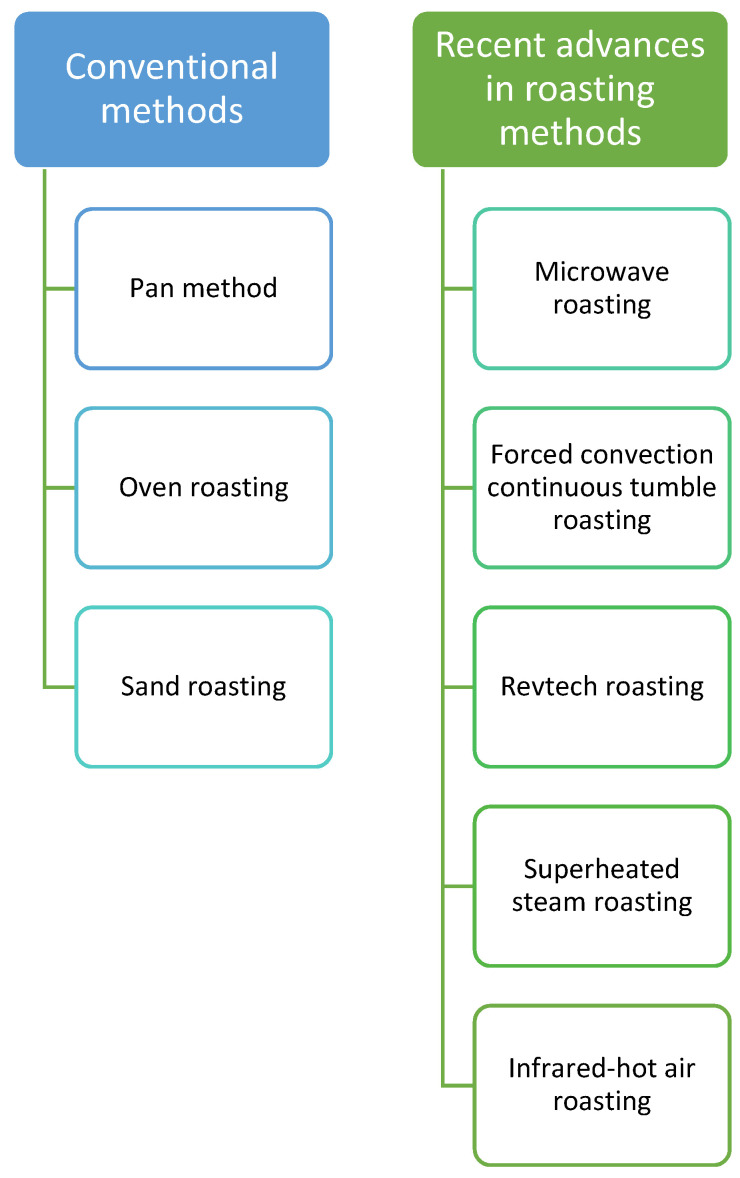
Conventional and emerging methods of roasting [109].

Roasting is an essential stage in coffee manufacturing because it enhances color, aroma, and flavor. The mechanism of heat transmission and the temperature profile used are the two most crucial processing characteristics that affect the physical and chemical qualities of roasted coffee. During roasting, physical changes in coffee beans are also technically significant, resulting in a significant drop in the expansion and density of the beans [107,108]. With the growing demand for fortified roasted goods and concerns about food safety and the impacts of hazardous chemicals, innovative roasting processes and equipment that transcend the limits of standard operations are required. A good overview of earlier work in the roasting method is given by Sruthi et al. [109]. They divided the roasting methods into two parts, based on conventional processes and recent advances in roast methods, respectively, as shown in Figure 3. The conventional methods consisted of techniques such as the pan method, oven roasting, and sand roasting. Recent advances in roasting techniques, such as microwave, revtech roasting, superheated steam roasting, forced-convection continuous tumble roasting, and infrared hot air roasting, are rapidly gaining popularity for their ability to successfully roast a variety of coffee types without sacrificing nutritional content [109].

There are several coffee-roasting technologies available in developing countries. Coffee beans are traditionally roasted in batch or continuous processes, depending on roasting procedures. Heat can be transmitted to the beans by conduction, when they directly contact hot metal surfaces, by free or forced convection caused by a streaming medium (hot air), or by radiation. The drum roaster is the most popular form of coffee roaster available for household and industrial usage. It is a revolving horizontal cylinder that roasts coffee beans placed inside it by continuously rotating and heating them with hot air pushed through the cylinder’s center or perforated sides, to achieve a uniform roast [110]. Fluidized bed-roasting is an innovative coffee roasting technique in which hot gas is pushed at the beans at a high velocity, often from the bottom of the roasting machine, the gases simultaneously heating and propelling the floating beans.

The latest study showed that the darker roasts resulted in the lowest concentrations of total chlorogenic acids and trigonelline in the coffee beans, and a 40%–60% drop in caffeine content was seen throughout the roasting process [110]. Drum roasting retains a higher amount of trigonelline in coffee beans than fluidized bed roasting or conventional roasting. Drum roasters may be the optimal solution for roasting specialty coffee beans without compromising their unique taste and fragrance. In their study, they investigated the influences of the drum, the fluidized bed, and traditional coffee roasting technologies on bioactive compounds of the Yirgacheffe, Harar, and Sidama varieties of specialty coffee beans cultivated in Ethiopia, studying these methods at light, medium, and dark roasting temperatures ranging from 150 °C to 200 °C for 7 to 15 min.

### 7.1. Effect of Roasting on Caffeine

The coffee species has a big impact on the caffeine level of green coffee. The caffeine levels of robusta coffees are greater than that of arabica coffees. Furthermore, the caffeine levels of most cultivars of C. arabica are believed to be comparable, but the caffeine concentrations of *C. canephora var. robusta* coffees vary widely [111,112].

Caffeine is moderately heat-stable during coffee roasting. However, due to sublimation loss at higher roast temperatures, caffeine concentration in dark-roast coffee tends to be lower than in lighter roasts [113].

A summary of caffeine compounds using various roasting methods is given in Table 4. In general, during the roasting process, a substantial decrease in the caffeine concentrations of the sample coffee beans was found. Similar work was also carried out by certain researchers [113,114,115]. Sidama coffee lost up to 60% of its caffeine content, while Yirgacheffe and Harar coffees lost an average of 53% and 40% of their caffeine content, respectively. This is owing to the fact that when the roasting temperature increases, the solubility of caffeine in water increases [116].

### 7.2. Effect of Roasting on Trigonelline

During coffee roasting, trigonelline, a pyridine derivative, is known to contribute indirectly to creating favorable taste compounds such as furans, pyrazine, alkylpyridines, and pyrroles. Trigonelline’s relevance has been extensively proven in earlier research as a precursor of taste and fragrance components and as a favorable nutritional element [111,117]. When trigonelline is heated in a sealed tube, nicotinic acid and nicotinamide and their O- and N-methyl derivatives are produced as reaction products, according to reports. In fact, investigations using mass spectrometry and NMR spectroscopy revealed that N-methylpyridinium and nicotinic acid are the main nonvolatile products of trigonelline pyrolysis [111,118,119]. NMR spectroscopy was used to study the time courses of trigonelline, N-methylpyridinium, and nicotinic acid.

Trigonelline, which is found in large concentrations in green coffee beans, is reduced constantly during the roasting process. Two thermal breakdown products of trigonelline, N-methylpyridinium and nicotinic acid, rose constantly throughout roasting, with N-methylpyridinium being the main thermal product. The drop in N-methylpyridinium concentration after 7 min of roasting was most likely due to additional breakdown and/or contact with other thermolytic products. The roasting degree was strongly associated with nicotinic acid, an essential vitamin and trigonelline’s second major thermal breakdown product. Other physicochemical aspects of a cup of coffee, such as flavor and fragrance, are certainly affected by trigonelline and its thermolytic metabolites, both directly and indirectly [111].

In line with the findings of this study, a review article reported a summary of trigonelline [110]. The medium-roasted Harar coffee had the greatest trigonelline concentration of 0.895% *w*/*w* when roasted in a drum roaster, followed by 0.855% when roasted in a fluidized bed-roaster, and 0.724% when roasted in a traditional or conventional oven top-roaster. Similarly, medium-roasted Yirgacheffee coffee had the greatest trigonelline concentration at 0.868% *w*/*w* when roasted in a drum roaster, followed by 0.756% *w*/*w* when roasted in a fluidized bed-roaster, and 0.639 percent when roasted in a conventional oven top-roaster. Medium-roasted Sidama coffee had the greatest trigonelline concentration, measuring 0.584% *w*/*w* when roasted in a drum roaster and 0.577% when roasted in a fluidized bed roaster. This suggests that drum roasting retains more trigonelline in coffees than a fluidized bed and conventional roasting. As a result, drum roasters may be the ideal option for roasting specialty coffee beans without compromising their unique aroma and flavor aroma [110].

### 7.3. Effect of Roasting on Chlorogenic Acid

Affeoylquinic acids (CQA), dicaffeoylquinic acids, feruloylquinic acids, and *p*-coumaroylquinic acids are among the 30 or more chlorogenic acids found in coffee beans [111,120]. As revealed by NMR spectroscopy, the amounts of CQAs fell significantly during roasting, but the levels of quinic acid, γ-quinide, and *syllo*-quinic acid decreased to a lesser extent. These findings are in line with those found in the literature, where the amount of chlorogenic acids in the sample coffee beans decreased continuously while they were roasted from raw to dark using a fluidized bed roaster. Both Harar and Sidama coffees demonstrated a 55% drop in their original chlorogenic acid concentration at a mild roasting temperature. Yirgacheffe, on the other hand, demonstrated just a 30% drop in chlorogenic acid content relative to its original amount. When the sample coffee beans were dark roasted, their chlorogenic acids levels decreased by an average of 90% [110].

**Figure 4:**
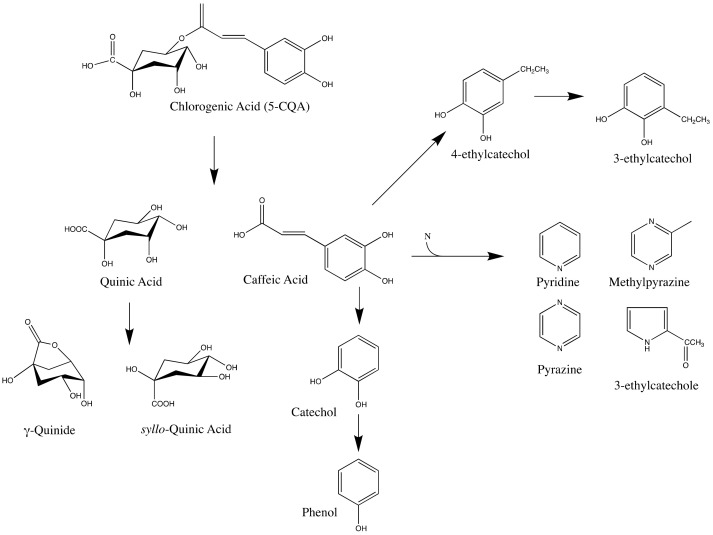
Changes of 5-caffeoylquinic acid during the roasting of coffee beans [112,121,124].

Chlorogenic acid breakdown might be utilized as a roasting degree indicator. Quinic acid is a prominent acid in green coffee beans and a result of chlorogenic acid breakdown (Figure 4). γ-quinide, an internal ester of quinic acid, was generated during the roasting process; *syllo*-quinic acid, another isomeric product of quinic acid, was also produced under common roasting circumstances, which included a thermal environment, low water content, and moderate acidity [111,121,122]. During the roasting of coffee beans, lactones of chlorogenic acids were discovered in addition to quinic acid lactones. Feruloylquinic acid lactones, caffeoylquinic acid lactones, and *p*-coumaroylquinic acid lactones were produced during coffee bean roasting, as indicated in the findings of Farah et al. [123] and their production was significantly dependent on roasting degree [123].

A light-medium roast is the best way to get the most lactones out of coffee, whereas deeper roasts give less [123]. Cinnamic acids are the other result of chlorogenic acids after roasting [125]. Cinnamic acids produced from chlorogenic acid breakdown might be involved in subsequent chemical processes that form other taste components.

### 7.4. Effect of Roasting on Total Phenolic Content (TPC)

Polyphenolic compounds are found in a variety of forms, including free, esterified, glycosidic, and insoluble-bound. The stability of polyphenols during heat processing is controlled by their structure and manner of interaction with the food matrix [109,126]. Numerous studies have established a trend in total phenolic content (TPC) caused by roasting.

A study discovered that roasted coffee samples at light, medium, and dark roasting levels had a lower TPC content than unroasted coffee [127]. Similarly, a drop in TPC was seen for arabica coffee roasted at 167, 175, and 171 °C [128]. Another study showed that TPC concentrations were affected by the degree of coffee roasting, which significantly impacts polyphenol content. When compared to lightly roasted coffee, polyphenol levels in highly roasted coffee decreased on average from 7.3% to 32.1% when utilized at industrial temperatures of 220 °C and a roasting duration of 17 to 24 min [129]. This event occurred as a result of a connection between TPC with thermal and oxidative degradation of these substances. Low molecular weight phenolic compounds readily volatilize at elevated temperatures. Additionally, polymerization and the formation of soluble chemicals such as melanoidins and polycyclic aromatic hydrocarbons have a detrimental effect on TPC. Highly intensive roasting procedures result in a greater loss of TPC due to the enhanced redox activity of polyphenols under such circumstances [129].

### 7.5. Effect of Roasting on Acrylamide Content

The amount of acrylamide in roasted coffee is regulated by concurrent creation and reduction processes that occur during roasting. Acrylamide production is predominant at the initial stage of the roasting cycle, resulting in elevated levels (>7 mg/kg) at this stage, which rapidly decline near the end of the roasting cycle due to quick degradation processes [111,120]. Over 95% of acrylamide is destroyed during roasting, as demonstrated by kinetic models and spiking studies using isotope labeled acrylamide. As a result, light-roasted coffees contain a higher concentration of acrylamide than extremely dark-roasted beans [111,120].

Increased roasting time promotes acrylamide destruction but also the production of unwanted off-flavor chemicals. As yet, no modification of roasting conditions has been carried out to reduce acrylamide production and maintain product quality [111,122]. Because coffee is a product with extremely specific quality characteristics, there currently appear to be few alternatives for mitigating acrylamide during roasting. Due to the fact that fragrance is a result of this heating process and is connected to the chemical components of the raw material, any change in the roasting parameters results in a different end product [111,123]. The significance of the roasting process for flavor and color and the relatively restricted variety of commercial goods complicate mitigation for coffee. Indeed, darker roasting as a possible method of reducing acrylamide may create additional unwanted chemicals, and would undoubtedly impair the batch’s flavor and aroma [130,131,132]. Therefore, no practical option exists for reducing acrylamide levels while maintaining the qualitative attributes of coffee, as the roasting procedure cannot be substantially altered [111,123].

A study shows the effect of coffee roasting procedures on the acrylamide level of roasted coffee [110], as shown in Table 5. The roast outcomes from each kind of roaster and degree of roast were significantly different in the experiments [110]. The greatest acrylamide concentration in Yirgacheffe coffee was produced at a light degree of roast for the drum and conventional roasters. However, the fluidized bed-roaster produced a greater acrylamide value in the coffee at a medium degree of roast. This might be attributed to the fluidized bed-roaster’s convective heat transmission, which results in roasted coffee with less volatile loss than coffees roasted in other types of roasters, as detailed by Nagaraju et al. [133]. The lowest levels of acrylamide were found at light roasting temperatures for fluidized bed-roasters, medium roasting temperatures for drum roasters, and dark roasting temperatures for conventional roasters. Fluidized bed-roasters produced the least acrylamide in the roasted samples of all the types of coffee roasters. As a result, a fluidized bed-roaster may be a superior option for producing low-acrylamide roasted coffee and lowering the risk of acrylamide poisoning in humans [110].

## 8. Storage of Roasted Coffee Bean

Several studies have found that acrylamide is not stable in commercialized coffee (beans and ground) when kept in its original container [111,134,135,136,137]. Coffees kept at room temperature for 6–12 months have lost 40–60% of their value [137]. After a three-month storage period at 10–12 °C, a study discovered a 30% decrease in acrylamide [134]. Another study examined the decrease in acrylamide in vacuum-packed coffee over 12 months at four different temperatures (−18, +4, ambient, and +37 °C). The rate of acrylamide depletion was shown to be strongly linked with temperature, with the greatest decrease rates occurring at 37 °C (>7-fold reduction in acrylamide levels after six months of storage time vs. starting concentration) [111].

## 9. Brewing

Brewing is the most common way to prepare roasted coffee beans for consumption. While there are numerous ways to brew coffee, nearly all require slowly infusing ground coffee beans with water [138,139]. Numerous variables must be considered for successful coffee brewing, as the process will affect the taste of the coffee and the active compound in it. The volatile ingredient is dissolved and lost during the brewing process, while the water-soluble component is dissolved in the water.

Temperature is a critical component in coffee brewing. Brewing can be performed by both cold and hot methods. These methods were compared by Rao et al. [140], who found that total antioxidant capacity (TAC) was only sensitive to the degree of roast in cold brew coffees, while hot brew coffees had a constant TAC for all three roast levels, and caffeine concentrations in cold brew samples were found to be similar to those in hot brew samples [140].

Nancy Cardoba et al. [141] also performed a study comparing hot and cold brew effects on the compounds in coffee. They found no significant variation in caffeine, trigonelline, or 4 and 5 caffeoylquinic acids between hot and cold brewing.

## 10. Conclusions

Changes in bioactive and chemical compounds occur in all phases of coffee processing. The quality of a beverage is affected by bean maturity, which is indicated by changes in the color of cherries. The accumulation of certain chemical compounds in mature coffee beans results in a flavorful beverage, and these compounds play an essential role in coffee processing.

The major bean components accumulate through ripening, and include cell wall polysaccharides, lipids, proteins, sucrose, and chlorogenic acids. However, the polyphenols chlorogenic acid and caffeoylquinic acid are detected in large amounts in green coffee beans. After harvesting ripe coffee cherries, coffee goes through many processes to become green coffee beans.

In general, three types of coffee processing procedures are used to produce green coffee beans, i.e., wet, dry, and semi-dry methods. Although coffee cherries may have the same maturity level before processing, the characteristics of coffee produced by dry and wet processing remain distinct. This is related to the germination process, which occurs immediately after depulping during the wet process. In contrast to the wet method, in which beans undergo a germination process, in the dry process, germination is inhibited and γ-aminobutyric acid (GABA) accumulates. Dry, wet, and semi-dry processing did not cause significant caffeine, chlorogenic acid, and trigonelline changes. Microbial fermentation mainly changes the structure of proteins and carbohydrates, resulting in metabolites that diffuse into the seeds to enhance flavor during roasting.

Environmental factors, physical conditions, and time all have an impact on storage. The use of a jute sack is recommended for storage of fewer than six months. Compared with HDPE plastic storage, the total phenolic and chlorogenic acid content in coffee stored using jute sacks decreased sharply at month ten. There was a significant difference in coffee stored in jute sacks for 11 months.

Roasting is an essential stage in coffee manufacturing because it produces color, aroma, and flavor. Caffeine is moderately heat-stable during coffee roasting. Trigonelline, which is found in large concentrations in green coffee beans, is reduced constantly during the roasting process. During roasting, chlorogenic acid levels decreases to 90%, depending on the roasting level. Chlorogenic acid breaks down into feruloyl quinic acid lactones, caffeoylquinic acid lactones, and p-coumarylquinic acid lactones, which produce the flavor.

## Figures and Tables

**Table 2:** Microorganisms isolated during coffee fermentation in dry, wet, or semi-dry processes.

Coffee Sample	Microbiota	References
*C. Arabica*, Dry process, 750–800 m above sea level Minas Gerais, Brazil	**Bacteria***: Acinetobacter* sp.; *Arthrobacter* sp.; ***Bacillus*** (*cereus*, *subtilis*, *macerans*, *polymyxa*, *megaterium*); *Enterobacter agglomerans*; *Yersinia* sp. **Yeast**: *Arxula adeninivorans*; *Candida* (*saitoana*, *fermentati*, *membranifaciens*); ***Debaryomyces*** (*polymorphus*, *Hansenii*); ***Pichia*** (*guilliermondii*, *guilliermondii*, *sydowiorum*, *subpelliculosa*, *burtonii*, *anomala*, *burtonii*); *Stephanoascus smithiae*; *Saccharomyces cerevisiae* **Fungi**: *Aspergillus* (*flavus*, *ochraceus*, *tamarii*, *niger*, *sydowii*); *Cladosporium cladosporioides*; *Penicillium* (*corylophilum*, *chrysogenum*, *brevicompactum*, *roqueforti*, *solitum*); *Fusarium solani*	[83]
*C. canephora*, Dry process, Jerônimo Monteiro 300 m above sea level	**Bacteria**: ***Bacillus*** (*cereus*, *licheniformis*, *pumilus*, *shackletonii*, *subtilis*); *Cellulosimicrobium cellulans*; *Citrobacter freundii*; *Enterobacter cloacae*; *Escherichia vulneris*; *Kosakonia cowanii*; *Lactobacillus* (*oligofermentans*, *oris*, *paracasei*); *Leuconostoc mesenteroides*; *Micrococcus* (*luteus*, *yunnanensis*); *Pantoea* (*agglomerans*, *ananatis*); *Staphylococcus* (*cohnii*, *epidermidis*, *haemolyticus*, *saprophyticus*, *xylosus*); *Stenotrophomonas maltophilia* **Yeast**: ***Candida*** (*glabrata*, *orthopsilosis*, *dubliniensis*, *parapsilosis*); *Meyerozyma guilliermondii*; ***Pichia cecembensis***	[89]
*C. canephora*, Dry process, Espírito Santo, Brazi l600 m above sea level	**Bacteria**: *Acinetobacter pittii*; *Acinetobacter radioresistens*; ***Bacillus*** (*altitudinis*, *cereus*, *safensis*, *subtilis*); *Citrobacter* (*braakii*, *freundii*); *Dermacoccus nishinomiyaensis*; *Enterobacter* (*asburiae*, *hormaechei*, *ludwigii*); *Enterococcus pallens*; *Enterobacteriaceae bacterium*; *Escherichia vulneris*; *Leuconostoc mesenteroides*; *Pantoea agglomerans*; *Pectobacterium parmentieri*; *Pseudomonas putida*; *Raoultella ornithinolytica*; *Salmonella* sp.; *Staphylococcus epidermidis*, *warneri*); *Streptomyces variabilis* **Yeast**: *Candida tropicalis*; *Hanseniaspora opuntiae*; *Hanseniaspora uvarum*; *Meyerozyma caribbica*; *Meyerozyma guilliermondii*; ***Pichia kluyveri***	[89]
*C. arabica*, Wet Processing 1300 m above sea level Jinghong in Yunnan, China	**Bacteria**: *Lactobacillus* (*coryniformis*, *plantarum*); ***Lactococcus*** (*hircilactis*, *lactis*); ***Leuconostoc*** (*citreum*, *holzapfelii*, *mesenteroides*, *pseudomesenteroides*); *Weissella soli* **Yeasts**: ***Candida*** (*humilis*, *quercitrusa*, *solani*); *Cordyceps brongniartii*; ***Hanseniaspora*** (*uvarum*, *vineae*); *Lachancea lanzarotensis*; *Papiliotrema terrestris*; ***Pichia kluyveri***; *Saccharomyces cerevisiae*; *Starmerella bacillaris*; *Torulaspora delbrueckii*; *Wickerhamomyces anomalus.*	[90]
*C. arabica*, Wet Processing 6.6 m above sea level Teven, NSW, Australia	**Bacteria**: ***Acinetobacter lwoffii***; ***Enterobacter ludwigii***; *Citrobacter koseri*; *Pseudomonas fluorescens*; *Klebsiella pneumoniae*; *Erwinia soli*; *Serratia marcescens*; *Brevibacillus parabrevis Anabaena*; *Salmonella enterica*; *Asaia* sp.; *Serratia marcescen*; *Brevibacillus parabrevis*; *Acetobacter persici*; *Gluconobacter cerinus*; *Leuconostoc mesenteroides*; *Lactococcus lactis* **Yeasts**: ***Hanseniaspora uvarum***; ***Pichia*** (*fermentans*, ***kudriavzevii***); ***Candida*** (*xylopsoci*, *railenensis*); *Wickerhamomyces anomalus*	[91]
*C. arabica* Semi-dry process, Minas Gerais, Brazi 750–800 m	**Bacteria**: *Weissella soil*; *Leuconostoc mesenteroides*; *Gluconobacter oxydans*; ***Enterobacter agglomerans***; *Leuconostoc mesenteroides*; *Erwinia* (*toletana*, *herbicola*); *Erwinia tasmaniensis*; *Klebsiella* (*oxytoca*, *pneumoniae*); *Pseudomonas aeruginosa*; *Morganella morganii*; *Acinetobacter* spp.; ***Bacillus*** (*cereus*, *macerans*, ***megaterium***, *subtilis*); ***Escherichia coli***; ***Lactobacillus*** (*brevis*, ***plantarum***); *Lactococcus lactis*; *Serratia* sp.; *Pantoea eucrina;* **Yeast**: *Arxula* sp.; *Candida* (*parapsilosis ernobii*, *fukuyamaensis*, *membranifaciens*, *carpophila*); ***Pichia*** (*guilliermondii*, ***anomala***, *caribbica*); *Saccharomyces* (*cerevisiae*, *bayanus*); *Debaryomyces hansenii*; *Mitchella repens*; *Trichosporon cavernicola*; *Rhodotorula mucilaginosa*; *Torulaspora delbrueckii.* **Fungi**: *Aspergillus* (*chevalieri*, *foetidius*, *niger*, *ochraceus*, *tubingensis*, *versicolor*); *Cladosporium* (*cladosporioides*, *macrocarpum*); *Cylindrocarpon* sp.; *Eurotium chevalieri*; *Fusariella* sp.; *Fusarium* sp.; *Fusarium* (*chlamydosporum*, *lateritium*, *nivale*, *solani*, *sporotrichioides*); *Geotrichum* sp.; *Mucor hiemalis*; *Penicillium* (*brevicompactum*, *commune*, *decumbens*, *fellutanum*, *implicatum*, *roqueforti*); *Phoma* sp.; *Ulocladium* sp.	[84,92]

Genus/Species with bold are Predominant species.

**Table 3:** The role of microorganisms during coffee fermentation.

Fermentation Impact	Microbiota	References
Pulp and mucilage degradation	**Bacteria**: *Bacillus*, *Aerobacter*, *Escherichia*, *Erwinia*, *Leuconostoc mesenteroides*, *Lactobacillus plantarum*, *Lactobacillus brevis*, *and Streptococcus faecalis* **Yeast**: *Candida* sp., *Pichia* sp., *Kluyveromyces* sp., *Schizosaccharomyces* sp., *Saccharomyces* sp., *Debaryomyces.*	[78,84,95,96,97]
Correlation with floral, fruity, and sweet character	**Yeast**: *Pichia* sp., *Saccharomyces cerevisiae.*	[92,98,99]
Produce organic acid	**Bacteria**: *Leuconostoc mesenteroides*; *Bacillus* sp. **Yeast**: *Candida parapsilosis*, *Saccharomyces cerevisiae.*	[92,98]
Inhibit ochratoxigenic fungi growth	**Yeast**: *Pichia kluyvery*, *P. anomala*, *Hanseniaspora uvarum*, *Leuconostoc* sp., *Weissella* sp., *Enterococcus.*	[78,94,100]
Produce mycotoxins and off-flavor	**Fungi**: *Aspergillus*, *Fusarium*, *and Penicillium*	[101,102,103]

Genus/Species with bold are Predominant species.

**Table 4:** Changes in caffeine levels during roasting-process of the various coffees grown in Ethiopia [110].

Roasting Method	Coffee Variety	Degree of Roast	Percentage (%)
Drum roaster	Yirgacheffe	Raw	1.572
		Light	0.722
		Medium	1.065
		Dark	0.887
	Harar	Raw	1.503
		Light	0.688
		Medium	0.876
		Dark	0.452
	Sidama	Raw	1.640
		Light	0.567
		Medium	0.567
		Dark	0.796
Fluidized bed roaster	Yirgacheffe	Raw	1.503
		Light	0.889
		Medium	0.885
		Dark	0.472
	Harar	Raw	1.640
		Light	0.842
		Medium	0.653
		Dark	0.470
	Sidama	Raw	1.572
		Light	0.637
		Medium	0.979
		Dark	0.935
Traditional roaster	Yirgacheffe	Raw	1.503
		Light	0.860
		Medium	0.687
		Dark	0.465
	Harar	Raw	1.572
		Light	1.478
		Medium	0.813
		Dark	0.997

**Table 5:** Changes in acrylamide levels during various roasting process of the different degrees of roasting [110].

Type of Roaster	Degree of Roast (mg/L)		
	Light	Medium	Dark
Drum	2.056	1.241	1.323
Fluidized bed	0.092	2.290	0.468
Traditional	2.351	1.068	0.702

## Data Availability

Available data are presented in the manuscript.
